# A lean method for selecting determinants when developing behavior change interventions

**DOI:** 10.1080/21642850.2023.2167719

**Published:** 2023-01-19

**Authors:** Rik Crutzen, Gjalt-Jorn Ygram Peters

**Affiliations:** a Department of Health Promotion, Maastricht University/CAPHRI, Maastricht, The Netherlands; b Department of Methods and Statistics, Faculty of Psychology, Open University of the Netherlands, Heerlen, The Netherlands

**Keywords:** determinants, behavior change, intervention development, formative research, methodology

## Abstract

When developing behavior change interventions in a systematic way, it is important to select determinants relevant to the target behavior. Data is needed to gain insight into the determinant structures (the relative strengths of associations between determinants and behavior) and their univariate distributions. This insight is crucial to select the most relevant determinants, but at the same time institutions tasked with behavior change (e.g. prevention organizations, municipal health services) often operate under prohibitive resource constraints, which also extend to how easily they can collect data from a sample. This paper introduces CIBERlite – an approach that furnishes the intervention developer with an idea of the relevance of a limited number of determinants using short measurements informed by theory. The first study (*N* = 401) in a series of three explores the convergent validity of short and full measurements of determinants derived from the Reasoned Action Approach. The short measurements are used in the main study (*N* = 415) that serves as a proof-of-concept for the CIBERlite plot, an efficient visualization combining data of determinant structures and their univariate distributions for eight behaviors. The unexpected patterns detected in the main study led to an expert estimation study (*N* = 45), which shows that individual experts have difficulty in predicting how people score on determinants. This stresses the importance of conducting determinant studies and CIBERlite is a valuable alternative to do so if resources are limited.

Behavior of people has an impact on their health and quality of life (Loef & Walach, 2012). So, supporting behavior change is essential in public health and to tackle other societal issues, such as mobility (Fujii & Taniguchi, 2006) and sustainability (Lo et al., 2012). When developing behavior change interventions in a systematic way, an important step is to first identify determinants relevant to the target behavior of an intervention (Bartholomew Eldredge et al., 2016). In such a problem-driven context, multiple theories are needed to gain insight into why people behave as they do. Subsequently, behavior change methods are selected that can be used in an intervention to target these determinants (Kok et al., 2016). These theories aimed at understanding behavior define these determinants and describe how they relate to behavior. For example, the Reasoned Action Approach (RAA) stipulates that attitude towards the behavior, perceived norms, and perceived behavioral control determine people’s intentions, while intention predicts behavior (Fishbein & Ajzen, 2010). RAA and other approaches stress that it is crucial to collect data regarding the behavior of interest in the population of interest. There are two reasons why this is important.

First, because the determinant structures might differ between behaviors. Determinant structures are the relative strengths of associations between determinants and behavior (e.g. as quantified by correlation coefficients). Fishbein and Ajzen (2010, pp. 190–191) mention the possibility to make predictions about these associations ‘on an intuitive basis or on the basis of other theories relevant to the behavior of interest.’ Such a basis could be differences between individuals. For example, attitudes might be associated more strongly with behavior than perceived norms for individuals that score low as opposed to high in self-monitoring tendency (Ajzen et al., 1982). Another basis could be differences between behaviors. For example,

it could be predicted that leisure behaviors, such as going to a movie, will be largely under the influence of attitudes, whereas behaviors that affect other people (e.g. littering) will show a stronger influence of perceived norms. Still other behaviors that involve serious potential barriers to their execution, such as quitting smoking cigarettes, may be expected to show evidence of the importance of perceived behavioral control (Fishbein & Ajzen, 2010, pp. 190–191).

There are studies available that focus on differences between behaviors in terms of their associations with possible determinants. For example, the contribution of perceived behavioral control to the prediction of ten different target behaviors varied inversely with the amount of control over the behavior (Madden et al., 1992). However, in general, assumptions about differences between behaviors are hardly tested in empirical studies.

Second, it is also important to collect data regarding the behavior of interest in the population of interest, because the univariate distributions of determinants might differ depending on the specific behavior or target group. For example, perceived behavioral control regarding smoking cessation is assumed to be low among smokers (Peters et al., 2018). However, often there is no data on univariate distributions of determinants for specific behaviors by specific populations in specific contexts. It is worthwhile, therefore, to establish whether a given population has relatively low or relatively high scores on measurement items for a given determinant. This can support determinant selection during intervention development. In addition, this can also be helpful to generate hypotheses and inform theory development.

## Selection of determinants

Insight into associations between determinants and the outcome of interest (e.g. behavior) and univariate distributions of determinants need to be combined in order to select relevant determinants. The need for selecting determinants stems from resources being finite, which has an impact on the quantity and quality of intervention content that can be (1) developed and (2) delivered. In order to make a selection of determinants to target, insights into (1) the associations between determinants and the outcome of interest need to be combined with (2) the univariate distributions. These two aspects are in line with the reasons to collect data regarding the behavior of interest in the population of interest.

The first is important because those determinants that have weak associations with the outcome will be less likely candidates to intervene upon: their low associations are taken as evidence of causal irrelevance.^1^ The second is important because it shows how much room for improvement there is to achieve the desired score on items related to a specific determinant. For example, from an informed-decision making point of view it might be worthwhile that smokers perceive the risks associated with smoking as applying to them. However, data on univariate distributions might show that most smokers do perceive these risks as applying to them, but perceived behavioral control to quit smoking is still low. In this example, perceived behavioral control will be a more viable intervention target as there is more room for improvement.

Confidence Interval-Based Estimation of Relevance (CIBER) is an approach to select determinants based on visualization of these two aspects: confidence intervals concerning both means of determinants and their correlations with behavior and/or more proximal determinants of behavior (Crutzen et al., 2017; Peters & Crutzen, 2018). Means and correlations are important parameters of, respectively, univariate distributions and the associations between determinants and the outcome of interest. Confidence intervals are used in the visualization of these parameters because they reflect the accuracy with which these parameters can be estimated based on available data (Peters & Crutzen, 2021). The use of visualization avoids the seeming accuracy and objectivity afforded by numbers (Peters, 2018). Moreover, visualization enables mapping the data onto spatial dimensions, facilitating comparison, which is necessary when making selections (in this case based on relevance of determinants).

Ranking determinants in terms of their relevance typically requires quantitative data (e.g. to produce CIBER plots). However, to develop the measurement instruments required for collecting this data, qualitative data on the beliefs underlying determinants is needed (as these are captured by items in the measurement instruments). Using empirical data and theory to achieve this can be complex and time-consuming (Ruiter & Crutzen, 2020). Sometimes intervention developers do not persevere in working through these difficulties (e.g. because of limited resources; Ten Hoor et al., 2022). In those cases, intervention developers are stuck with developing behavior change interventions based on what they *think* are relevant determinants regarding the behavior of interest in the population of interest. This incomplete understanding of determinants might —even with good intentions paving the road— result in ineffective solutions and wasted resources. In the worst-case scenario, the solutions might even be counterproductive.

## The CIBERlite approach

The current paper proposes a compromise between, on the one hand, thoroughly going through the complex and time-consuming process described above, and on the other hand, collecting no data at all. This compromise is a lean method for selecting determinants when developing behavior change interventions, based on CIBER. The proposed approach is called CIBERlite and aims to furnish the intervention developer with an idea of the relevance of a limited number of determinants using short measurements informed by theory. In other words, it is an approach to be used in circumstances where resources are limited.

In the current paper, we use RAA as the theoretical framework, because it is a commonly used approach to understanding health behaviors (McEachan et al., 2016) with standardized direct measurements (Fishbein & Ajzen, 2010). In short, the RAA states that intention – the readiness to engage in a behavior – is the most important predictor of behavior. Intention is shaped by three determinants: attitude, perceived norm, and perceived behavioral control. Attitude is a latent disposition or tendency to respond with some degree of favorableness or unfavorableness to the target behavior, based on perceived usefulness of that behavior to achieve one’s goals (instrumental attitude) and what one expects to experience if engaging in that behavior (experiential attitude). Perceived norm concerns the perceived social pressure to perform (or not perform) a behavior. This is based on perceptions concerning what should or ought to be done with respect to performing a given behavior (injunctive norm) and whether others are or are not performing the behavior (descriptive norm). Perceived behavioral control concerns people’s perceptions of the degree to which they are capable of, or have control over, performing a given behavior – referred to as capacity and autonomy respectively. The determinants as specified in the RAA (attitude, perceived norm, and perceived behavioral control) are used for demonstrative purposes. In other words, the inclusion of determinants one decides to measure when applying the CIBERlite approach is guided by theory – in this case RAA. We would like to stress that CIBERlite is not theory-specific and can be used to compare the relevance of a limited set of determinants from other theories as well. This paper aims to demonstrate a lean method to gain insight into the relevance of attitude, perceived norm and perceived behavioral control in the prediction of intention to perform various behaviors as well as the univariate distributions of these determinants.

**Figure 1.**
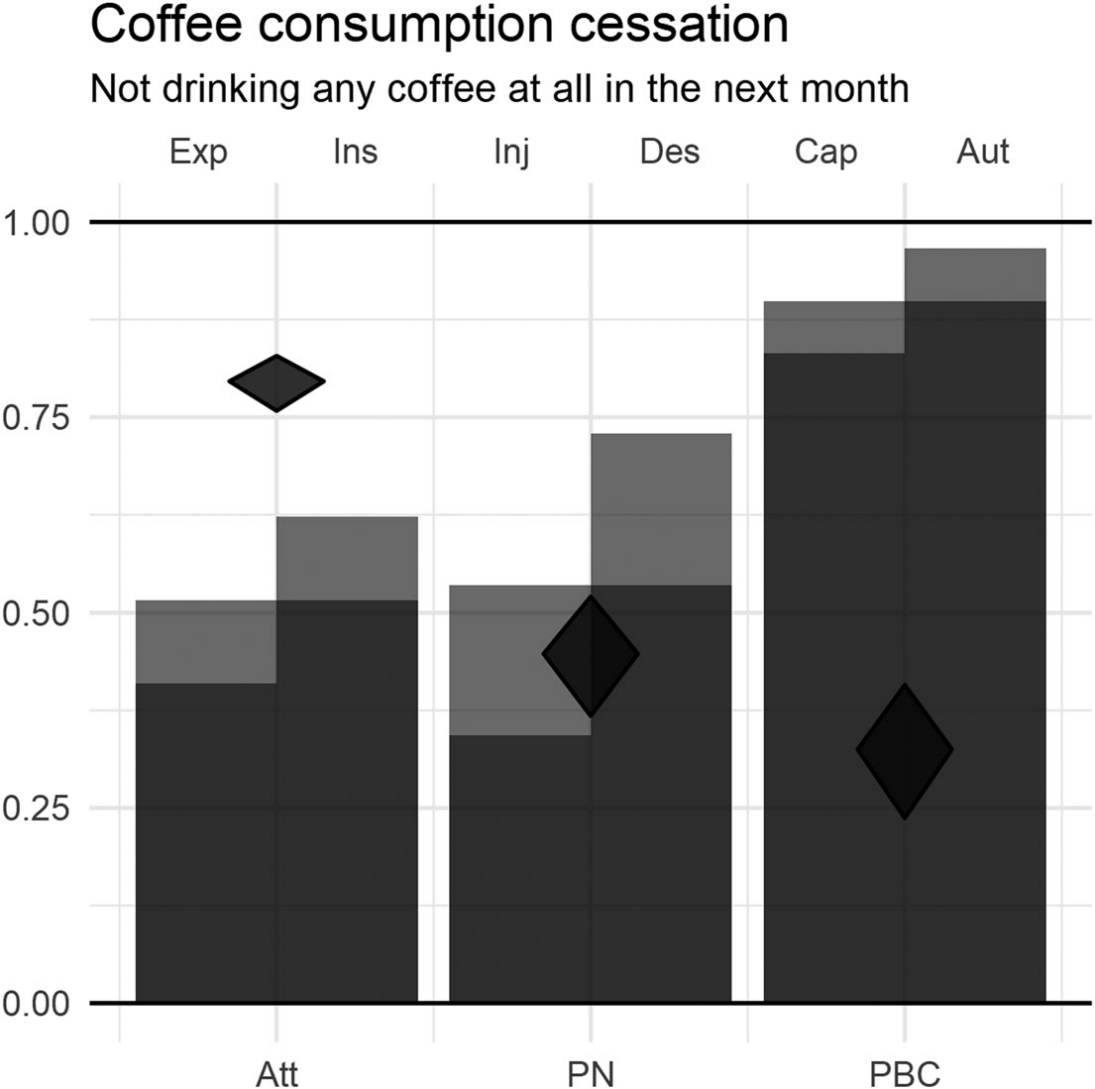
An example of a CIBERlite plot. Note. Bars represent means of experiential (Exp) and instrumental (Ins) attitude (Att); injunctive (Inj) and descriptive (Des) perceived norm (PN); and capacity (Cap) and autonomy (Aut) regarding perceived behavioral control (PBC). Diamonds represent (confidence intervals of) correlations between determinants and intention.

The core component of CIBERlite is the CIBERlite plot (see Figure 1 for an example on coffee consumption cessation). This data visualization can be considered a ‘light’ version of the Confidence Interval-Based Estimation of Relevance (CIBER) plot: it also presents information about determinants’ univariate distributions and their associations to a given target (in this case, behavioral intention). The diamonds represent the confidence intervals for the correlation coefficients between the determinants and the target. The narrow vertical bars represent the means for the more specific determinants, labeled at the top axis (experiential attitude, instrumental attitude, injunctive norms, descriptive norms, capacity, and autonomy), and the broader vertical bars represent the means for the overarching generic determinants (attitude, perceived norm, and perceived behavioral control), labeled at the bottom axis. The light gray area of the narrow bars visualizes how the means of the more specific determinants differ from the mean of their overarching generic determinant. In Figure 1, the bars represent the determinants as specified in the RAA, but it is a generic template that can be adapted. When using the Extended Parallel Process Model as a theoretical framework (Witte, 1992), for example, the component of threat could be represented by two narrow vertical bars for susceptibility and severity and the component of efficacy could be presented by two narrow vertical bars for self-efficacy and response efficacy. When using the Self-Determination Theory (Deci & Ryan, 2002), the different types of motivations could be represented by different bars.

Conceptually, the CIBERlite plot differs from the CIBER plot in three ways. First, CIBERlite plots are grayscale, forgoing the explanatory role of color so as to not overload the visualization with information. Second, CIBERlite plots do not show the raw data points, again in an attempt to make the plots more accessible (and less intimidating to users without a strong quantitative background). Third, CIBERlite plots are optimized for showing information about determinants, whereas CIBER plots are optimized for showing information about subdeterminants (i.e. more lowel-level constructs specified for a given behavior, population, and context, such as specific advantages of forgoing coffee for a month that are nonsensical in the context of other behaviors such as seatbelt use).

This example CIBERlite plot in Figure 1 (see the Methods section of the Main study for more details on how data was collected) shows that the means of attitude and perceived norm are similar (with instrumental attitude having a higher sample mean than experiential attitude, and descriptive norm having a higher sample mean than injunctive norm), whereas the mean of perceived behavioral control is considerably higher. The correlation of attitude with intention is higher than that of perceived norm, which is higher again than that of perceived behavioral control. In this scenario, assuming that a higher mean indicates the desired value, an intervention developer struggling with limited resources may decide to focus mostly on attitudes (specifically, experiential attitudes, as these have the lowest mean and therefore most room for improvement). The intervention developer would also notice the high mean for perceived behavioral control, which might suggest people overestimate how easily they can ‘kick the habit’ of drinking coffee.

The second component to the CIBERlite approach is a lean measure for a set of determinants. The CIBERlite plot shown above shows the results at item-level: this plot can be obtained after presenting seven items to a sample.^2^ The underlying reasoning for this drive for brevity is again that it acknowledges the resource constraints that institutions tasked with behavior change (e.g. prevention organizations, municipal health services) operate under, which also extend to how easily they can collect data from a sample. By using a small set of items, they obtain maximum insights with minimal investment; a balance that we hope can help persuade them to consistently engage in this minimal determinant study. To illustrate the CIBERlite plot, we needed a brief set of items fit for the purpose, that we refer to as the RAA CIBERlite items.

## Study 1: Convergent validity of RAA CIBERlite items

In the main study reported in this paper, we used two items to assess people’s attitude, two items for perceived norm, two items for perceived behavioral control and one item for intention (hereafter: the short version – see Table 1). These items were chosen from a larger set of items per determinant that is recommended when using direct measurement within RAA (Fishbein & Ajzen, 2010; hereafter: the full version). For example, when measuring attitude in the full version, there are two items regarding instrumental attitude and two items regarding experiential attitude. From these four items, two were chosen, such that one item concerned instrumental attitude and one item concerned experiential attitude (we attempted to select the items that most closely reflected each construct’s definition). The results of this main study were very surprising in terms of the determinants’ central tendencies not being as expected. However, we realized that despite the short version being a subset of the full version, that was no absolute guarantee of convergent validity of the short version and the full version. We wanted to rule out the possibility that patterns we observed in the main study would reflect invalid measurement of the relevant constructs rather than determinant structures. Therefore, the aim of this study is to explore the convergent validity of short and full measurements of these determinants. Although this study was conducted *after* data collection for the main study had already started (which we also acknowledge in the pre-registration), we switched the order of reporting in this paper to reflect the logical order in which these studies should have been conducted.

**Table 1.** RAA CIBERlite items.

Determinants		Items	Response scale anchors	
Intention		I intend to do X	Absolutely not	Absolutely
Attitude	Instrumental	For me, X is …	Bad	Good
	Experiential	For me, X is …	Unpleasant	Pleasant
Perceived norm	Injunctive	If I do X, then most people that are important to me would … of this	Disapprove	Approve
	Descriptive	How many people like you do X?	Nobody	Everybody
Perceived behavioral control	Capacity	I’m confident that, if I want to, I can do X	No confidence at all	Very confident
	Autonomy	Whether I do X is …	Not up to me	Completely up to me

*Note. ‘*X’ is a placeholder for the behavior of interest.

### Methods

For each of five behaviors separately, participants randomly received either the full version or a short version of items to assess the determinants of these behaviors. The study was pre-registered at the Open Science Framework (OSF): https://osf.io/hvkzr. Furthermore, all materials used in this study (e.g. the survey) as well as non-identifiable data, analysis scripts, and output of the analyses are available at https://osf.io/pemfz (note that we froze the project’s contents using OSFs ‘registration’ functionality, to preserve its state upon manuscript submission and safeguard against future changes in the associated GitLab repository; see https://osf.io/5btha for that registration). These efforts are taken to acknowledge a call for full disclosure to maximize scrutiny, foster accurate replication, and facilitate future data syntheses (e.g. meta-analyses; Crutzen et al., 2012; Peters et al., 2012).

#### Ethics statement

Ethical approval was granted by the Research Ethics Committee of the Open University of the Netherlands (approval number: U2017/03081/FRO).

#### Participants and procedure

We used an online survey to collect data among students at the Open University of the Netherlands. The only selection criterion is that participants need to be at least 18 years of age. No payment for participation was awarded, but students could earn course credit by participating in the online survey. Participants could indicate whether they want their data to be deleted and we share (and used) only data from participants that have consented to this. Data collection took place between May 2018 and January 2019.

#### Measurements

Data were collected regarding five different behaviors:

- Not drinking any alcohol at all in the next month
- Not drinking any coffee at all in the next month
- Not smoking at all in the next month
- Exercising at least one hour every week in the next month
- Finishing a marathon in the next month.

All participants answered questions about all behaviors. To eliminate order effects, the order of behaviors was randomized in the online survey (by LimeSurvey). Intention, attitude, perceived norm, and perceived behavioral control were assessed for each of these behaviors. The items for the short version are shown in Table 1. In the full version, all determinants were assessed by the four items recommended by the RAA (Fishbein & Ajzen, 2010). The response scale that was used for all these items was a 5-point Likert scale, instead of a 7-point Likert scale as used in RAA. This decision was based on previous evidence rejecting 7 scale points as an optimal number and indicating that the added value of having more than 5 scale points is generally little (Lissitz & Green, 1975) and may even yield data of lower quality (Revilla et al., 2014).

All these items had to be completed, meaning that there is no missing data unless participants drop out. After completing these items for all behaviors, participants were asked whether they additionally would like to complete items regarding socio-demographics: age (open text field), sex (female; male; other …), educational level (primary school; intermediate secondary education; higher secondary education/preparatory university education; intermediate vocational education; higher vocational education; university; other …), and the first two digits of their zip code to assess urbanity versus rurality. Collection of data on socio-demographics was optional to implement data minimization (Crutzen et al., 2019).

#### Sample size justification

We are interested in the convergence of indices based on short and full versions of operationalizations to assess the determinants. We used the tables provided by Moinester and Gottfried (2014) to estimate sample size for correlations with pre-specified confidence intervals. Based on *r *= .80 and half-width *w *= .05, the required sample size is 205 participants. Taking into account wider confidence intervals, this sample size is also sufficient for *r *= .55 and *w *= .10. Taking into account possible missing data (e.g. because participants stop before finishing the study), we aimed to recruit 250 participants.

#### Analyses

First, before assessing the internal structure regarding measurements of determinants for five behaviors, we have verified dimensionality by means of exploratory factor analyses (Crutzen & Peters, 2017) and using eigenvalues for the full version. The eigenvalue is a measure of how much of the variance in the observed variables a factor explains. Any factor with an eigenvalue >1 explains more variance than a single observed variable (Kaiser, 1960). Second, to estimate internal consistency, we have calculated McDonald’s *ω* (a less biased alternative to coefficient *α*) for the full version and the Spearman-Brown coefficient (*ρ*) for the short version.^3^ Third, we have calculated correlations (*r*) between all determinants for the full version and the short version separately. Finally, to explore the convergent validity between these two versions, we have calculated *Q* to assess differences between these correlations for full and short versions (Cohen, 1992).

In general, we report confidence intervals of effect sizes to focus on accuracy of reported estimates instead of drawing attention to specificity of point estimates. Given the relative width of most sampling distributions and the subsequent variation that occurs in estimates over samples (Moinester & Gottfried, 2014; Peters & Crutzen, 2021), caution in basing decisions on the exact computed numbers seems prudent. Given the large number of reported confidence intervals, we use a higher confidence level than commonly used (i.e. 99% instead of 95%) for the key estimates in this study (i.e. *r*, *Q*). Moreover, we visualize these estimates to forego the seeming accuracy and objectivity afforded by numbers. More specifically, confidence intervals are represented using the diamond shapes commonly used for the aggregated effect size in meta-analyses. Unlike error bars with whiskers, diamonds do not draw attention to the confidence interval bounds (Peters, 2018). More detailed output of the analyses is available at https://osf.io/pemfz.

### Results

In total, 501 participants that were willing to share their data initiated the survey and 401 completed the survey (completion rate: 80%). Of those that completed items regarding socio-demographics, the average age was 36 (*Q_1 _*= 26; *Q_3 _*= 43) and 78% was female. The vast majority (93%) was highly educated (i.e. higher vocational education or university) and 23% came from a (very) rural area (both according to categorization of Statistics Netherlands).

Table 2 shows the results of the first two steps of the analyses.^4^ As a first step, with regard to dimensionality, eigenvalues of the first two factors are reported for each determinant of all five behaviors. Eigenvalues are >1 for all first factors. For the second factor, 18 out of 20 eigenvalues are <1 and two are >1. For the latter two, however, there is a relatively sharp drop in eigenvalues of the first and the second factor, and as everything computed from a sample, eigenvalues are subject to error, with point estimates varying from sample to sample, and any single estimate being either too high or too low. Given this random variation, these patterns warrant extracting one factor for each determinant of all five behaviors. As a second step, with regard to internal structure, values of *ω* and *ρ* regarding intention, attitude and perceived behavioral control were relatively high. The only exception was perceived behavioral control for running a marathon, where the short version scored low. For perceived norm, the short version scored relatively low for all behaviors, reflecting the underlying formative measurement model.

**Table 2.** Dimensionality and internal structure for measurements of determinants.

Determinant	Behavior	Full version			Short version
		Eigenvalues		ω	*ρ*
		Factor 1	Factor 2		
Intention	Alcohol	3.30	0.44	[.91; .95]	-^a^
	Coffee	3.54	0.29	[.95; .97]	-^a^
	Smoking	3.25	0.47	[.88; .93]	-^a^
	Exercising	3.75	0.13	[.97; .98]	-^a^
	Marathon	3.27	0.53	[.85; .91]	-^a^
Attitude	Alcohol	2.42	0.93	[.67; .79]	.72
	Coffee	2.54	0.76	[.77; .86]	.86
	Smoking	2.40	0.89	[.54; .70]	.63
	Exercising	2.72	0.67	[.81; .88]	.62
	Marathon	2.79	0.73	[.82; .89]	.72
Perceived norms	Alcohol	1.79	0.91	[.50; .69]	.51
	Coffee	1.77	1.02	[.47; .66]	.33
	Smoking	2.20	0.86	[.70; .82]	.25
	Exercising	2.01	0.86	[.63; .76]	.19
	Marathon	1.95	0.89	[.44; .64]	.28
Perceived behavioral control	Alcohol	2.85	0.65	[.84; .90]	.54
	Coffee	2.45	0.81	[.81; .88]	.64
	Smoking	3.07	0.60	[.89; .93]	.79
	Exercising	2.44	0.85	[.74; .83]	.62
	Marathon	2.01	1.24	[.61; .76]	.34

Note. Eigenvalues of first two factors and 95% confidence intervals for *ω* for full version; and Spearman-Brown (*ρ*) for short version.

^a^ Not applicable because in the short version intention was assessed by one item only.

**Figure 2.**
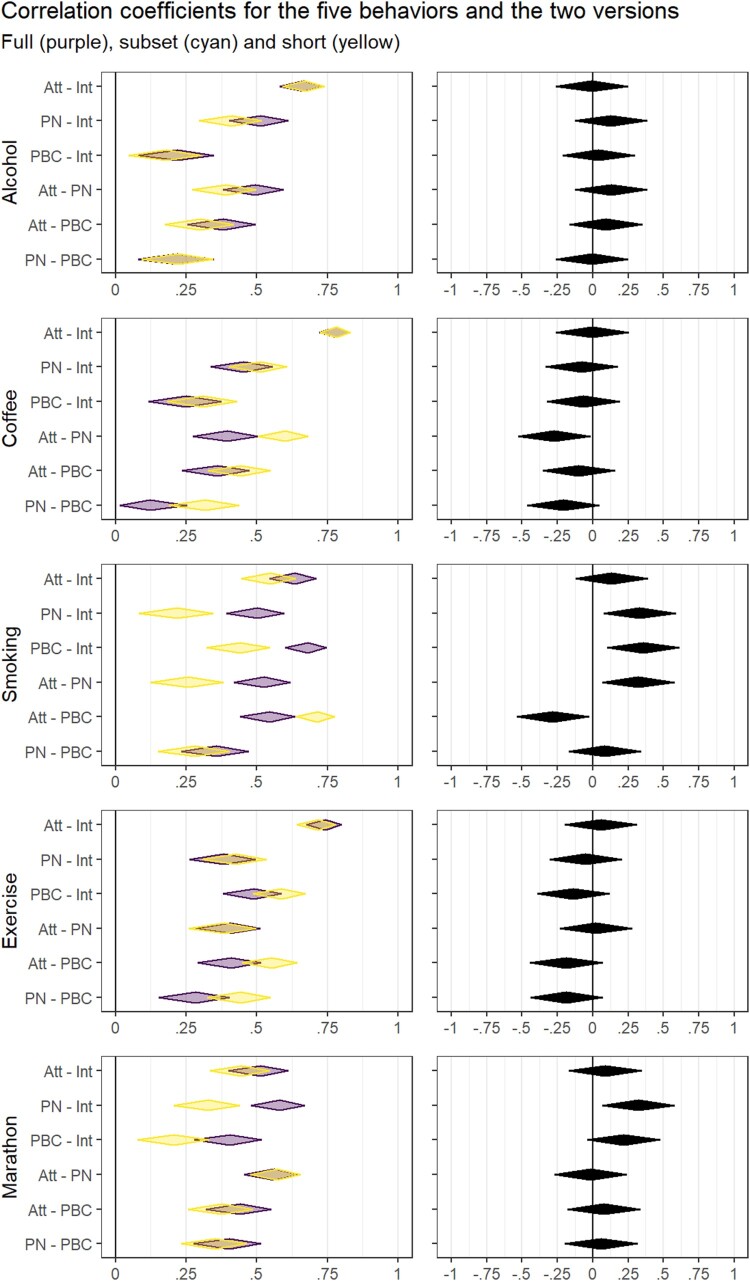
Correlations between determinants and differences between full and short versions. Note. Left panel shows correlations (*r*) between intention (Int), attitude (Att), perceived norm (PN), and perceived behavioral control (PBC) for full version (purple) and short version (yellow). Right panel shows differences between these correlations for full and short versions (*Q*).

Figure 2 shows the results of the more informative^5^ final two steps of the analyses. First, the left panel shows correlations between intention, attitude, perceived norm, and perceived behavioral control for both versions. Second, the right panel shows differences between correlations for full and short versions by means of diamond plots representing *Q*. In general, the confidence intervals of *Q* included zero for most determinants of all behaviors, except smoking. For smoking, for four out of six correlations between determinants, there was a difference between the full and the short version. These differences were small, and may reflect sampling variability – but they may also reflect heterogeneity in participants’ response processes for each of the four RAA items.

In short, this study demonstrated sufficient convergent validity of the short version of operationalizations to assess the determinants of these behaviors: these seemed mostly appropriate to be used in the main study.

## Study 2: Main study

The main study focuses on a lean method to support evidence-based selection of determinants (in this study, attitude, perceived norm and perceived behavioral control) in intervention development. This selection is based on the determinants’ associations with intention to perform various behaviors as well as the univariate distributions of these determinants. To obtain a proof-of-concept for the CIBERlite plot, an efficient visualization of the determinant structure of a given behavior, we measured the behavioral determinants for eight behaviors that we selected to vary systematically in attitude, perceived norm, and perceived behavioral control.

### Methods

A full factorial 2 × 2 × 2 within-subjects (i.e. repeated measures concerning determinants of eight behaviors) design with three dependent variables (i.e. the determinants attitude, perceived norm, and perceived behavioral control) was used in this study. The study was pre-registered at OSF: https://osf.io/cjz6n. Furthermore, all materials used in this study (e.g. the survey) as well as non-identifiable data, analysis scripts, and output of the analyses are available at https://osf.io/2uwxp.

#### Ethics statement

Ethical approval was granted by the Research Ethics Committee of the Open University of the Netherlands (approval number: U2017/03081/FRO).

#### Participants and procedure

Participants and procedure were similar to the study on convergent validity. Data collection took place between January 2018 and November 2018.

#### Measurements

Data were collected regarding eight different behaviors. We selected the following behaviors, based on our expectation for the determinants’ central tendencies. The conditions they represent in the 2 × 2 × 2 design are listed in parentheses, where ‘Att’ stands for Attitude, ‘PN’ stands for Perceived Norm, ‘PBC’ stands for Perceived Behavioral Norm, and ‘lo’ and ‘hi’ indicating, respectively, that we expect –as specified in the pre-registration– most people in the general population to exhibit relatively low or high scores on that determinant.

- Not drinking any coffee at all for the next month (Att: lo, PN: lo, PBC: lo)
- Mainly traveling by public transport for the next month (Att: lo, PN: hi, PBC: lo)
- Sometimes running a red light (by bike) in the next month (Att: lo, PN: lo, PBC: hi)
- Always carefully separating waste in the next month (Att: lo, PN: hi, PBC: hi)
- Con somebody for 10.000 euro in the next month (Att: hi, PN: lo, PBC: lo)
- Finish a marathon in the next month (Att: hi, PN: hi, PBC: lo)
- Often take extra long showers in the next month (Att: hi, PN: lo, PBC: hi)
- Brush your teeth every day in the next month (Att: hi, PN: hi, PBC: hi)

All participants received all conditions (i.e. answered questions about all behaviors). To eliminate order effects, the order of behaviors was randomized in the online survey (by LimeSurvey).

We used the RAA CIBERlite items in Table 1. More details on Spearman-Brown coefficients can be found at https://osf.io/mxncy. The response scale that was used for all these items was a 5-point Likert scale. All these items had to be completed, meaning that there is no missing data unless participants drop-out. After completing these items for all behaviors, similar to the previous study, participants were asked whether they additionally would like to complete items regarding socio-demographics: age, sex, educational level, and the first two digits of their zip code to assess urbanity versus rurality.

#### Sample size justification

We aimed for achieving 95% power to detect a small effect size (*f* = .1) within a full factorial 2 × 2 × 2 within subjects design. We expected the repeated measures (i.e. the measures of the three determinants attitude, perceived norm, and perceived behavioral control for each of the eight behaviors) to correlate weakly (i.e. *r* = .1), and because each factor (low vs. high expected attitude, low vs. high expected perceived norm, and low vs. high perceived behavioral control) has only two levels, sphericity is guaranteed. As the three dependent variables are analyzed separately, we have used an alpha of .005/3 = .0016667. G*Power (Faul et al., 2007) was used to conduct the power analyses, which resulted in a required sample size of 391 participants. This coincides nicely with the required sample size to get reasonably accurate estimates of the population correlation coefficients for the association between the three determinants and intention for each behavior (to obtain a margin of error (confidence interval half-width) of .1, even for a correlation as low as *r* = .1, 383 participants suffice) (Moinester & Gottfried, 2014).

#### Analyses

We produced CIBERlite plots to visualize means of determinants (in this case six items related to attitude, perceived norm and perceived behavioral control) and the association of these determinants with the outcome of interest (in this case intention to engage in eight different behaviors). The CIBERlite plot has been implemented in the **behaviorchange** R package (Peters et al., 2022) in the **CIBERlite()** function. Its main arguments are ‘data’ (to pass the data frame), ‘determinants’ (to pass the column names of the determinants), and ‘targets’ (to pass one or more targets, such as e.g. intention). The function produces the plot in Figure 1, which can be further customized by adding, for example, a subtitle (as done in Figure 1).

We also conducted three full factorial 2 × 2 × 2 repeated measures analyses of variance to test for differences in the determinants’ means, with our expectations regarding attitude, perceived norms, and perceived behavioral control (low versus high) as factors, and the scores on these three determinants as dependent variables. The full factorial analyses resulted in seven main ‘effects’ and interaction terms for each of the three determinants, so 21 main ‘effects’ and interaction terms in total. In addition, we have conducted descriptive and exploratory analyses, which can be consulted in the rendered R Markdown file available at https://osf.io/kte98.

### Results

In total, 514 participants that were willing to share their data initiated the survey and 415 completed the survey (completion rate: 81%). Of those that completed items regarding socio-demographics, the average age was 36 (*Q_1 _*= 27; *Q_3 _*= 44) and 79% was female. The vast majority (89%) was highly educated (i.e. higher vocational education or university) and 21% came from a (very) rural area (both according to categorization of Statistics Netherlands).

**Figure 3.**
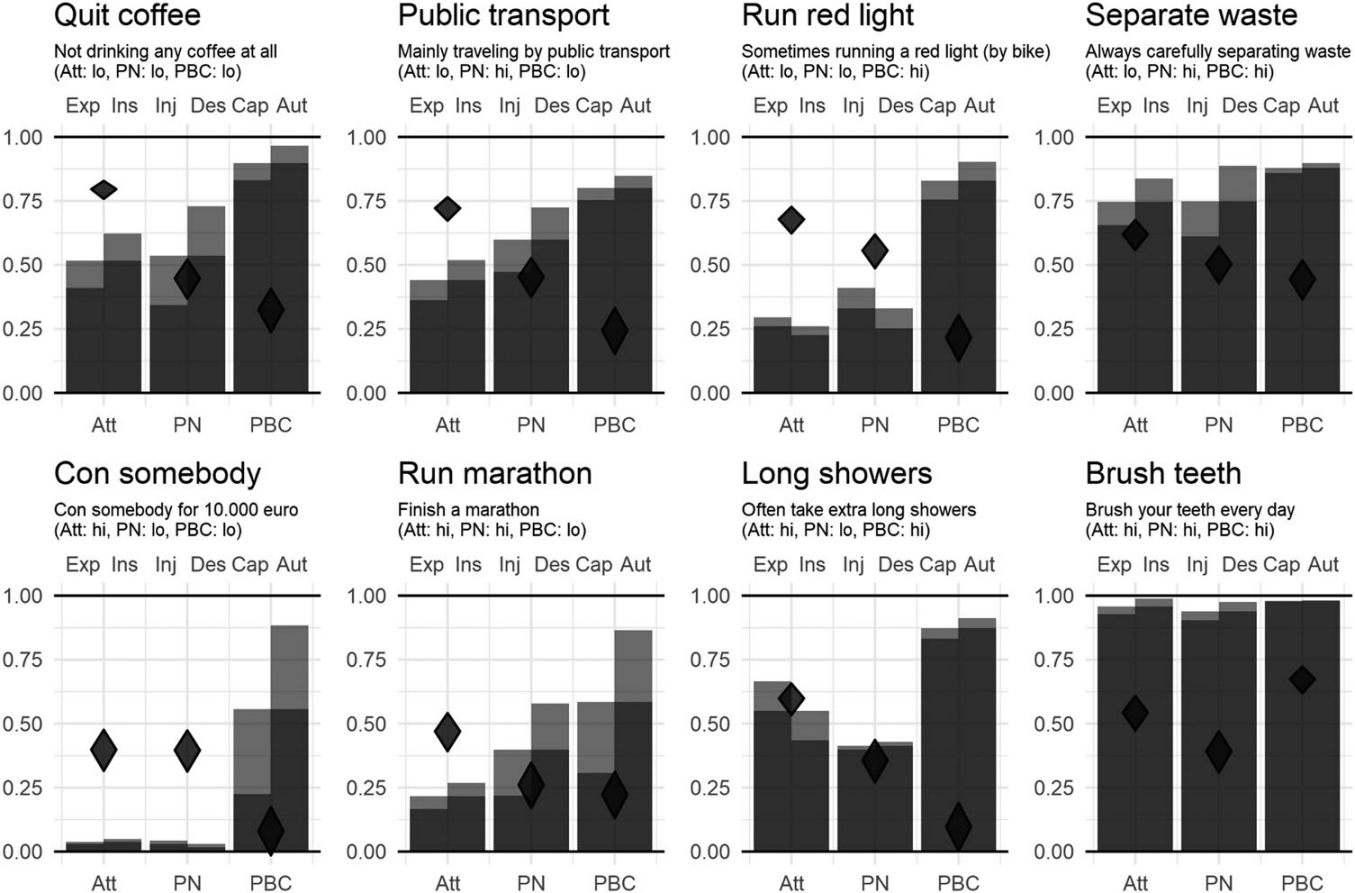
CIBERlite plots for eight different behaviors. Note. Bars represent means of experiential (Exp) and instrumental (Ins) attitude (Att); injunctive (Inj) and descriptive (Des) perceived norm (PN); and capacity (Cap) and autonomy (Aut) regarding perceived behavioral control (PBC). Diamonds represent correlations between determinants and intention.

Figure 3 shows the means of six items related to attitude, perceived norm and perceived behavioral control and the association of these determinants with intention to engage in eight different behaviors. These eight CIBERlite plots are combined in one figure that provides a comprehensive overview allowing simultaneous evaluation of a large amount of information. This also facilitates comparison between CIBERlite plots. The figure also shows that the values of the means were not as expected. For example, perceived behavioral control to travel by public transport scored high, but the association with intention to do so was lower in comparison with attitude. In this example, attitude might be a more relevant intervention target in comparison with perceived behavioral control. There is more room for improvement, in comparison with perceived behavioral control, and it is stronger associated with the outcome of interest.

The planned 2 × 2 × 2 factorial repeated measures ANOVA showed that the *p*-values for all 21 main ‘effects’ and interaction terms were lower than 10^−7^, except for the association of our prior expectation of low perceived norms with reported perceived behavioral control (*p* = .0003) and the three-way interaction between our prior expectations and reported perceived behavioral control (*p* = .002). However, the unexpected patterns in the means indicates that these results are not straightforward to interpret.

When substantively interpreting the CIBERlite plots, one striking result is the single behavior where results matched our predictions: almost all participants reported high attitude, norms, and PBC for brushing their teeth every day. For this behavior, the few participants reporting lower values also had lower intentions, resulting in relatively high associations. Despite these associations, the CIBERlite plot clearly shows that none of these three determinants seem promising intervention targets: there is no room for improvement.^6^ Another striking result is conning somebody, where there is a large difference between autonomy and capacity, two determinants constituting PBC. Like for running a marathon, participants believe this behavior to be under their control, but do not believe they have all required subskills. For attitude and norms, conning somebody shows the opposite pattern as teeth brushing: both are low, with correlations driven by the participants reporting high values.

## Study 3: Expert estimation

Confrontation with these unexpected patterns prompted a period of self-reflection as to our perceived expertise in the behavior change domain. We could see three straightforward explanations for these patterns. First, perhaps the short measures were not valid. However, the results from the study into the short measures’ convergent validity suggest that while some attenuation of patterns might reasonably be expected, such radically different patterns as we observed seem unlikely. This left two explanations. One explanation was that perhaps we had not accumulated as much behavior change expertise as we had hoped, and these results reflect our failure to somewhat accurately predict whether people have low or high attitudes, perceived norms, and perceived behavioral control given a specific behavior,. The last remaining explanation was that estimating attitudes, perceived norms, and perceived behavioral control for a given behavior is virtually impossible even for behavior change experts.

Establishing which of these explanations is the more likely one is important, because if the last explanation is plausible, determinant studies fulfill an irreplaceable function in intervention development endeavors. In that case, confidence that experts can fruitfully reason about whether a target population is likely to be highly self-efficacious, about whether there exist strong norms to engage in a behavior, or about whether people are generally positively or negatively inclined towards a behavior seems unwarranted. Instead of relying on such a rationalist epistemological perspective, resorting to empiricist alternatives seems inevitable.

In that scenario, if behavior change science is to progress towards a future where it does have a solid basis for reasoned conjectures about determinants, first a frame of reference for such conjectures must be obtained. Such a frame of reference requires descriptive evidence as to the determinants for many behaviors and in many populations and contexts. However, at present, anecdotal evidence from our experience and that of our colleagues suggests that journals often reject determinant studies that can furnish us with exactly that evidence, based on the argument that such studies do not directly test a theory. While that observation is true, ironically, the results of Study 2 suggest that that position may hamper scientific progress. Because of these potentially far-reaching implications, we conducted a third study to explore how accurately a convenience sample of behavior change experts could predict determinants for these eight behaviors.

### Methods

To explore whether behavior change experts could reliably estimate whether a population with specific characteristics has high or low attitudes, perceived norms, and perceived behavioral control for a range of behaviors, we used a convenience sample recruited through our professional networks. The study was pre-registered at OSF: https://osf.io/fzgm8. Furthermore, all materials used in this study (e.g. the survey) as well as non-identifiable data, analysis scripts, and output of the analyses are available at https://osf.io/rwvsx.

#### Sampling strategy, procedure, measurements, and analysis plan

The final sample consisted of 45 participants, described more in detail in the results section. They participated anonymously in a single survey. The survey first described the aims of the study. Then, the target population was described, specifically, the population of undergraduate students in the Netherlands from which we sampled in the main study. This was followed by an example of the exercise that would follow. In the actual exercise, on three consecutive pages, participants rated their expected attitude, perceived norms, and perceived behavioral control for each of the eight behaviors in three array questions (with the behaviors in the rows as subquestions, and the five possible scores (1–5) in the columns; see the survey at https://osf.io/rwvsx for more details). Finally, participants were asked about their behavior change experience with three questions (their role in terms of behavior change expertise; their experience with the RAA; and the number of years they have studied behavior change). To analyze the data, we planned to compute means and standard deviations for each of the 24 estimated parameters.^7^ We consider confidence intervals containing values below 0.5 as indications that the population standard deviation may well be trivial.

#### Sample size justification

In line with the study’s focus on accuracy, we used an accuracy in parameter estimation approach to compute the required sample size. We aimed to estimate the standard deviation in the expert estimates with sufficient accuracy. To estimate a standard deviation of at least 0.7 with sufficiently tight confidence intervals (specifically, with a half-width of 0.3), we computed that we would require at least 14 participants (note that confidence intervals get wider as standard deviation point estimates increase, but as the point estimates increase as well, the confidence intervals’ lower bounds will also increase). We set as a stopping rule that we would stop data collection at the first of May 2019, unless our sample would contain fewer than 14 participants at that point, in which case we would continue until we reach 14 participants (see the pre-registration at https://osf.io/fzgm8). If we had more participants by May 2019, then this would allow us to explore inter-group differences.

### Results

Of the 45 participants, 22 identified as researchers who studied behavior change (e.g. employed by a university); 10 as somebody who did research and also developed interventions; 1 as a practitioner who worked with behavior change (e.g. intervention developer); 8 as none of the above (e.g. when your job doesn’t involve applying or doing behavior change science); and 4 as something else. In terms of experience with the RAA, 16 participants had used the RAA or its predecessors in practice or research, but not for a determinant study; 13 had heard of the RAA or its predecessors, but never worked with it; 9 had used the RAA or its predecessors for one or more determinant studies; and 7 had never heard of the RAA or its predecessors. Behavior change experience varied from 0 to 15 years, with a mean of 4.6 years, a median of 3 years, a mode of 2 years, and and a standard deviation of 4.1 years.

#### Variability in expert estimates

The lower bounds for the confidence intervals for the standard deviations for the 24 estimates varied from 0.5 (for the estimated attitude regarding conning somebody) to 1.3 (for the estimated perceived behavioral control regarding conning somebody). All lower bounds exceeded 0.5, our predefined threshold value for considering negligible variance in the population realistic: we conclude that it is exceedingly unlikely that experts converge in their estimates of people’s determinants (i.e. whether for a given behavior, people would have high versus low attitudes, or norms, of perceived control). As an indication of how much variation they exhibit, the upper bounds for the standard deviation confidence intervals varied from 0.8–2.0 (for the same two estimates that had the lowest confidence interval bounds). Variance was highest for perceived behavioral control estimates: the lower bounds for five out of eight confidence intervals exceeded 1.0. The mean standard deviation was 1.04; the mean confidence interval lower bound was 0.86, and the mean upper bound was 1.31.

#### Accuracy of expert estimates

**Figure 4.**
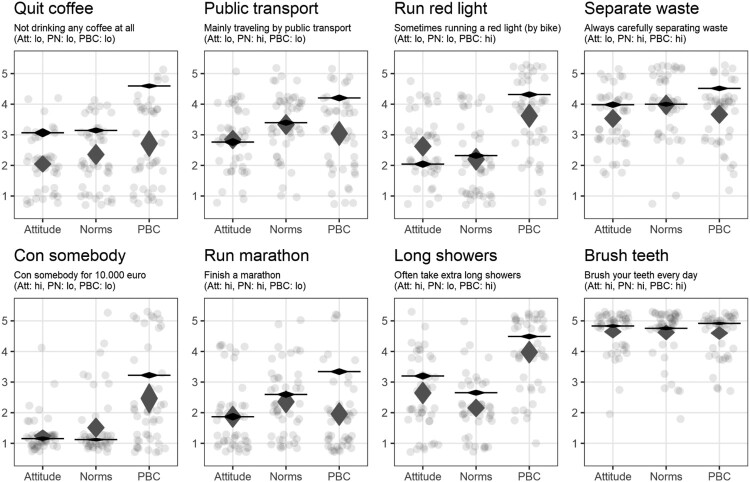
The expert estimates and their means, as well as the means for each determinant based on the main study. Note. The grey diamonds represent means of expert estimates of attitude (Att), perceived norm (PN) and perceived behavioral control (PBC). The dots represent estimates of individual experts with jitter added to prevent overplotting. The black diamonds and horizontal lines represent means of participants in the main study (similar to Figure 3).

Despite substantial heterogeneity in expert estimates, their mean estimates were relatively accurate. Figure 4 shows every expert estimate, as well as the 95% confidence intervals for the mean estimates of these experts, and including the means for each determinant based on the data from participants in the main study (i.e. undergraduate students). This suggests that while single experts are unable to accurately predict whether determinants will be high or low, if estimates are obtained from sufficient experts, their aggregate provides a reasonable approximation of the actual values.

#### Variability by expertise

To explore whether variability was smaller for participants with the same role, smaller for participants with the same number of years of experience with behavior change science, or smaller for participants with similar experience with the RAA, we also looked at standard deviation estimates in those subgroups. In these subgroups, because of the smaller sample sizes, the standard deviation confidence intervals are wider, trivially resulting in both less high lower bounds and higher upper bounds. The mean standard deviation was around 1.0 (i.e. one-fifth of the full scale range) in every group. Mean lower bounds ranged from 0.6 for the 7 participants with 10 or more years of experience with behavior change to 0.8 for the 25 participants with 4 or less years of experience with behavior change. Mean upper bounds ranged from 1.4 for the 22 participants who identified as having the role of a researcher who studies behavior change tot 4.1 for the 4 participants with a ‘other’ role. No systematic differences seemed to emerge: it was not the case that one or more subgroups had notably higher convergence or divergence in expert estimates (see the R Markdown file with the full analyses at https://osf.io/rwvsx for all standard deviations and confidence interval bounds).

## Discussion

In this paper, we present a set of three studies that originated from a project designed to illustrate CIBERlite, a lean method to estimate relative determinant relevance. We conceived of CIBERlite based on our observation that institutions tasked with behavior change (e.g. prevention organizations, municipal health services) often lack the resources and time, and sometimes the expertise, to conduct the extensive multimethod determinant studies required as a prerequisite to developing effective interventions. However, the results of the two additional studies that we ended up doing besides the main study suggest a much broader need for wide-scale determinant studies.

We examined (and confirmed) the convergent validity of the lean CIBERlite measures with the full measures as recommended by the original authors of RAA (Fishbein & Ajzen, 2010). In the main study, we presented the univariate results for the determinants for eight target behaviors, selected based on our expectation that they would systematically vary in the mean levels of attitude, perceived norm, and perceived behavioral control in the population. Although these levels did vary, the patterns were not as predicted. To eliminate the possibility that our inability to predict determinant levels reflected insufficient behavior change expertise, we explored the variability in the estimates of these determinants in a wider convenience sample of behavior change experts, and found considerable variability that did not seem to be negatively associated with self-reported expertise.

From a more overarching point of view, we consider the findings of the expert estimation study – showing that individual experts have difficulty in predicting how people score on determinants – to be very thought-provoking. At this stage, it is unclear what may explain this substantial variability. We consider not only the heterogeneity in estimates striking, but also how closely the aggregated estimates approximate the means from the main study sample. At the same time, this proximity may suggest an explanation: it could be that experts have (unconsciously) based their ratings regarding expected attitude, perceived norms, and perceived behavioral control scores for each of the eight behaviors on their own beliefs. This is an interesting avenue for future research and at the same time stresses the importance of conducting determinant studies for future practice. The main study shows that ‘educated guesses’ by individual experts are so erratic that they can hardly serve as a stand-in for even a lean empirical approach to gain insight into determinants. This implies that intervention developers cannot solely rely on individual experts – well-intentioned though they may be – lest they risk serious consequences (i.e. ineffective solutions and wasted resources). The gold standard remains making a full inventory of all possible determinants using the core processes (Ruiter & Crutzen, 2020). However, CIBERlite is a valuable alternative if resources are limited. So, it is not a replacement, but an alternative that is better than not conducting a determinant study and solely relying on expertise. It is an accessible first step to move from a rationalist epistemological perspective to an empiricist one.

### Limitations

Two possible limitations need to be mentioned with regard to the studies presented in this paper. First, the study on convergent validity of the short version of operationalizations to assess determinants showed that these were mostly appropriate to be used in the main study. However, for smoking behavior, there were differences in the full and the short version in terms of correlations between determinants, which would make the short version for determinants of smoking behavior less appropriate. Although smoking behavior was not part of the main study, which was initiated before the convergent validity study, this warrants further exploration. If participants’ response processes for the different items exhibit a higher degree of heterogeneity for smoking, which characteristics of this behavior cause this? The results of the study on convergent validity also imply that when it is needed to develop similar brief sets of items to assess constructs from other theories that contain less items than recommended by such a theory, that it is warranted to first explore convergent validity.

Second, this study uses two samples consisting of undergraduate students only, which were slightly older than average because of the specific university setting that was used for recruitment. The Dutch government’s purpose in founding the Open University of the Netherlands was to make higher education accessible to anyone with the necessary aptitudes and interests, regardless of formal qualifications. This is reflected, for example, in the average age of participants in this study being higher than the average of ‘typical’ undergraduate students that started their studies after high school. This does not allow generalization of its findings to other populations and contexts. However, the focus was on showing the added value of a lean method to gain insight into the relevance of determinants, based on their association with behavior and their univariate distributions. This method can be applied across behaviors, populations, and contexts.

### Practical recommendations

A practical recommendation is a plea for widespread publication of determinant studies. Clearly, even experts with ample theoretical training lack sufficient substantive context to reliably estimate whether a given population will be high or low on attitude, subjective norms, and perceived behavioral control – and there is no reason to assume they will fare better for determinants from other theories. To remedy this, broad mapping and publication of determinant structures for different behaviors, populations, and contexts can provide a frame of reference that can ultimately equip experts with firm grounding for such conjectures. To achieve this, descriptive research seems called for, specifically, determinant studies reporting univariate distributions, ideally with the same measures across behaviors, populations, and contexts to enable comparison. A pragmatically positive corollary is that to map univariate distributions, even data collected by observational cross-sectional designs can be highly valuable. Synthesizing these yields a strong evidence base for a comprehensive understanding of people’s dispositions regarding a wide range of behaviors in various contexts. We hope CIBERlite can facilitate such large-scale determinant studies.

### Conclusion

This paper presents two useful tools to facilitate determinant studies – even if resources are limited. First, the RAA CIBERlite items that can be used across behaviors, populations, and contexts. We would like to reiterate that CIBERlite is not theory-specific and we stimulate efforts to develop similar brief sets of items to assess constructs from other theories. Second, the possibility to visualize data in the CIBERlite plots by means of the **CIBERlite()** function that is open source and freely available (Peters et al., 2022).

## Notes

1 This requires the questionable assumption of ergodicity: that between-person associations are informative about within-person associations, and by implication, processes (Fisher et al., 2018). When inferences about within-person associations based on between-person associations does not seem reasonable, then unless resources allow employing intensive longitudinal designs, inspection of determinants’ univariate distributions becomes the main empirical basis for determinant selection decisions.

2 Technically, there are no limits in the numbers of items that can be presented in a CIBERlite plot – as in, such a plot can be used to visualize anywhere from one to dozens of determinants. However, with too many determinants, the plot will lose its value as a lean method to select determinants, as it might become overwhelming. In that case, alternative means of visualization become more functional (e.g., CIBER plots).

3 Perceived norm has a formative rather than reflective measurement model, which means these internal consistency measures enable comparison to other studies, but cannot estimate reliability (Gruijters et al., 2021).

4 We were amongst those pleading for these first two steps of the analyses (Crutzen & Peters, 2017). However, we have since learned that these steps may fall short of providing the kinds of rigorous, potentially falsifying tests of relevant hypotheses commonly expected in scientific research. Maul (2017) illustrates this via a series of studies in which participants were presented with survey items deliberately constructed to be uninterpretable, but the application of these steps nonetheless returned favorable-appearing results.

5 See previous footnote.

6 For those few participants who *do* report lower attitudes, norms, and PBC, there *is* room for improvement. However, health promotion interventions often have to make decisions based on what can achieve the most effect in their population of interest as a whole.

7 In the pre-registration, we indicated we also planned to compute correlations. In hindsight, we are not able to say how this aligns with the aim of this study.

## Open Scholarship

This article has earned the Center for Open Science badges for Open Data, Open Materials and Preregistered. The data and materials are openly accessible at Study 1: https://osf.io/pemfz, Study 2: https://osf.io/2uwxp and Study 3: https://osf.io/rwvsx. The preregistrations are openly accesible at Study 1: https://osf.io/hvkzr, Study 2: https://osf.io/cjz6n and Study 3: https://osf.io/fzgm8.
